# Analysis of the Patients Hospitalised in Paediatric Trauma Centers in Poland in 2019

**DOI:** 10.34763/jmotherandchild.20212503SI.d-21-00027

**Published:** 2022-02-09

**Authors:** Ewa A. Biegańska, Jan Stachurski, Karol Rokicki

**Affiliations:** 1 Student Research Association of Paediatric Emergency Medicine, Medical University of Warsaw Warsaw, Poland; 2 Department of Emergency Medical Services, Faculty of Health Sciences, Medical University of Warsaw Warsaw, Poland

**Keywords:** Trauma centers, emergencies, emergency treatment, paediatric emergency medicine, advanced trauma life support, emergency room

## Abstract

**Background:**

Paediatric trauma centers (PTCs) are facilities that were established to provide traumatised patients with fast, accurate diagnoses and optimal treatments. In Poland, they have been functioning since 2017. Our research aimed to assess the overall activity of the PTCs and cross-sectional data of their patients in Poland in 2019.

**Material and methods:**

We have analysed data provided by the National Health Fund (NHF) about the activity of seven trauma centers for children. For the PTC in the Paediatric Teaching Clinical Hospital University Clinical Center of the Medical University of Warsaw, we have gathered the data from the internal documentation system.

**Results:**

In Poland, in 2019, there were seven operating PTCs. During that year, they hospitalised 195 severely traumatised patients. The available data have shown that no specialised tracking system of children admitted to PTCs was used; we have obtained data reporting final diagnoses, not the preliminary ones. Summarising the data from the PTC in Warsaw, in the first year of its operation there were 32 patients admitted, of which only 8 have met the criteria of admission.

**Conclusions:**

Due to the small number of patients reported, it is difficult to draw specific conclusions about the efficacy of PTCs in Poland. Obtaining reliable data is difficult, as there is no paediatric trauma patients database. To assess and improve the quality of PTCs, it would be profitable to create a national system monitoring the events and collecting data on the treatment results.

## Background

Injuries are among the most significant causes of morbidity and mortality worldwide, among all ages of patients [1, 2, 3]. They are, however, the most common cause of death and disability in paediatric patients, aged from 1 to 19/18 years [1, 2, 4]. It has been previously highlighted in Polish literature, that external causes account for more than 50% of deaths in paediatric and adolescent age group, being in fact the first cause of death in men up to 39 years of age and women up to 24 years of age. They are also the leading cause of hospitalisation among children and adolescents. [5] Moreover, according to Eurostat calculations, the risk to life due to accidents in Poland in 2016 was almost 10 % higher than the average in the whole EU. [5] Although the downward trend has been observed since 2000, the rate of change was markedly slower in Poland than in other European countries such as Luxemburg or Denmark, and as a result the estimated excess mortality rate for external causes increased more than four-fold between 2000 and 2013. [6, 7] The fact that injuries are such a burden for health of children and adolescents strongly motivates healthcare systems to look for systemic solutions and to improve care for those patients. Currently, in many countries specialised trauma centers have been organised both for adult and for paediatric patients, which should provide them with fast, reliable and detailed diagnosis, and with therapy protocols of highest quality, resulting eventually in better outcomes. Based on existing analyses, it is evident that in Poland it is particularly important to appropriately organise care for trauma patients in the youngest age group. As for now, such trauma centers have existed since 2017 and are functionally distinct from the general hospitals. Children qualified for trauma care are admitted to the hospital emergency departments apart from the general unit, and also separately financed.

Our paper aims to review the overall activity of paediatric trauma centers (PTCs) in Poland in 2019 and to analyse patients from the Trauma Center operating under the Emergency Department in Paediatric Teaching Clinical Hospital University Clinical Center of the Medical University of Warsaw, in 2019 – the first year of its existence. We are interested in investigating how many severe paediatric trauma patients are hospitalised in Poland, what diagnoses are the most common, and whether there are nationwide statistics that gather data concerning trauma patients. In addition, we want to examine the long- and short-term morbidity and mortality of this group of patients.

## Material and methods

Ethics committee approval was received for this study from the Ethics Committee of the Medical University of Warsaw – decision number AKBE/93/2021.

Data on formal requirements to be met by PTCs and the patients admitted come from the Regulation of the Minister of Health of 25 January 2016 on Trauma Centers for Children, with later changes. [8]

To obtain nationwide data on services in trauma centers for children in 2019, we made an official request to the headquarters of the National Health Fund (NHF) for the list of those facilities, the number of children admitted, broken down by voivodeships and the age of patients, the most common diagnoses, the most frequently performed procedures and the number of deceased patients.

For more detailed analysis of PTC medical services, we have used data provided by the Emergency Department in Paediatric Teaching Clinical Hospital University Clinical Center of the Medical University of Warsaw.

We have then proceeded to detailed analyses of the paediatric patients for the following general information: sex, age, meeting the conditions for admission to the center, the mechanism of the injury, forms of transportation, number of surgical interventions, time in which the surgery was performed, type of surgical intervention, admissions to intensive care unit (ICU) and duration of the ICU stay, duration of general hospitalisation and the direction of discharge from the hospital.

In both cases, the statistical data were processed using Microsoft Excel 365. The https://www.calcmaps.com website was used to prepare the maps.

## Results

### The data from National Health Fund (NHF) database concerning all national paediatric centers at the time of the analysis

According to the NHF, in 2019, there were 7 hospitals reporting patients hospitalised in PTCs, and such data were analysed. However, for scientific integrity it should be noted that by 2021, 3 more trauma centers for children were opened, which gives a total of 10 trauma centers for children at the time of submitting the article. [9]

The data obtained from the NHF have not given direct information about patients admitted to the trauma centers. The information we were able to obtain came from the screening of patient data from hospitals with trauma centers, basing on the reported diagnoses, which should be possible to report only in facilities with a signed contract for the trauma center.

By analysing the national data that we have eventually managed to obtain from NHF, it turned out that in 2019 a total of 195 paediatric patients, with 62% of them being males and with an average age of 11, were hospitalised in trauma centers, with the highest number of hospitalisations, 59, in the Lublin Province. One patient was hospitalised twice, so the total number has increased to 196.

The data obtained by us have shown that the most common diagnoses were those in the field of head trauma, with epidural hemorrhage, traumatic brain edema, and concussion accounting for 15.4%, 9.2%, and 9.2 % of diagnoses respectively. This also explains the fact that the most often performed surgical procedures were those in the field of neurosurgery – craniotomies and skull trepanations. The most common procedure was general tracheal anesthesia. Of all trauma patients admitted to PTCs, less than a half (36.7%) were admitted to intensive care units, and 2 patients died within 30 days of hospitalisation. Hospitalisation time for 40% of patients was up to 7 days, for 23.5% from 14 to 30 days and 12.5% of the patients were hospitalised over 30 days.

**Figure 1:**
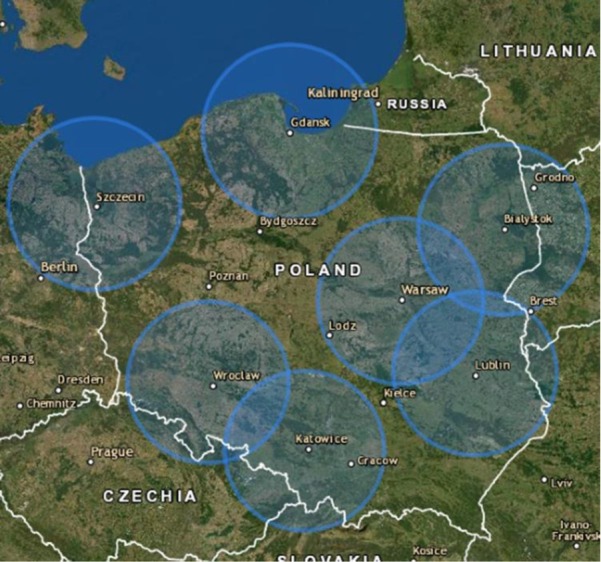
Map of Poland representing the zones that allow reaching the 7 trauma centers operating in 2019 from the site of the event within the statutory 90 minutes.

When analysing the location of trauma centers operating in 2019, we also noticed that a significant part of Poland is outside the zone that allows reaching the trauma center from the site of the event within the statutory 90 minutes [Fig. 1].

## The data from the trauma center operating under the Emergency Department in Paediatric Teaching Clinical Hospital University Clinical Center of the Medical University of Warsaw

Summarising the data from the paediatric trauma center in Warsaw during the first year of its operation, there were 32 patients admitted. The majority (81%, 23/32) were males, and the average age was 10.6, which corresponded quite well with the national data. In the analysis of the data, we have found out that car strikes, road traffic crashes, and falls from heights were the most common events leading to the injury. Surgical intervention was required by 43% (14 out of 32) patients, and 2 children required 2 interventions. The most common procedures were orthopedic, followed by neurosurgical: 8 and 4 out of 14, respectively. Of these interventions, 8 out of 14 were performed within 8 hours of admission. When analysing the groups of patients in terms of the mechanism of injury, we have noticed that the highest number of surgical interventions was performed in the car strike group, while in the group of falls from heights no surgical intervention was needed [Table 1]. After analysing detailed case reports, only 8 out of 32 patients met the criteria for admission to the trauma center. For eligible patients, the most common injury mechanism was a fall from a height followed by a car strike: 5 and 2 out of 8, respectively. Of the patients who met the admission criteria, only 2 required surgical interventions. When comparing the two groups of patients, the one that met the criteria and one that did not, we noticed that in the latter group the number of surgical interventions was significantly increased, while in the group of patients who met the criteria, the frequency of hospitalisation in the ICU was higher and the mean duration of stay there was longer [Table 2]. Moreover, when analysing the patients in terms of individual groups of criteria, it has turned out that 7 out of 24 children from the group without fulfilled criteria did meet the anatomical criteria but did not meet the physiological ones.

**Table 1:** Comparison between groups of patients with different mechanisms of trauma.

Cause of trauma	Total number	Number of patients with fulfilled criteria	Number of surgical interventions	Number of intensive care admissions	Mean hospitalisation time (days)
Fall from height	5	5	0	0	8,9
Road traffic crash	5	0	2 (40%)	0	11
Car hit	8	2	6 (75%)	2 (25%)	11
Others	14	1	6 (43%)	1 (7%)	9,35

**Table 2:** Comparison between the groups of patients who did and patients who did not fulfill the criteria for PTC admission.

Groups of patients	Number of patients	Number of surgical interventions	Number of surgical interventions performed in the first 8 hours after admission	Number of Intensive Care admissions	Mean time of hospitalisation (days)
Patients with fulfilled criteria	8	2 (25%)	1 (12.5%)	2 (25%)	11.875 (days )
Patients without fulfilled criteria	24	12 (50%)	7 (29.2%)	1 (4.2%)	8.33 (days)

Most of the patients were discharged home, one to another hospital, two to rehabilitation and care centers, and one patient has died. One of the patients discharged to the rehabilitation center, as well as the patient who has died, were from the group in which the patients fulfilled admission criteria.

## Discussion

Severe injuries are most significant cause of morbidity and mortality in the paediatric population. Apart from that, children also present many distinct features in their physiology and anatomy, which make them more sensitive to high-energy traumas. Based on these differences, it was concluded that perhaps the paediatric population would benefit from the creation of dedicated, child-only trauma centers, rather than admitting children to adult trauma centers. After establishing the separate trauma centers for children, it was found that centralisation of care for paediatric trauma patients resulted in their increased survival and has provided more favorable care for this group. According to research conducted in the USA and Japan, establishing specialised trauma centers for children resulted in faster transport to a place with appropriate resources to provide treatment and quick diagnostics; this has consequently improved patients’ survival. [10, 11, 12, 13, 14, 15, 16, 17] Some authors suggest that better outcomes, lower hospital mortality and morbidity are especially significant in younger children. [18] Others argue that the different type of center does not in fact improve mortality rates of the patients; however, the care is optimised in PTCs [19]. One of the reasons for a better chance of survival in paediatric and mixed trauma centers could be the under-triage of paediatric trauma patients in facilities for adults. [14]

The experiences of other countries have prepared the ground for establishing separate organisational units in the form of trauma centers for children in the Polish health care system. What is more, it has been previously suggested that because of high mortality and morbidity rates related to injuries, and the high rate of excess mortality among paediatric patients in Poland, such organised care for trauma patients is especially needed. [6, 7]

According to the Polish ministry of health, there are 17 adult trauma centers and 8 paediatric trauma centers operating in Poland currently and 5 new PTCs are planned to be opened. [9, 20]

To be qualified for admission and treatment in a paediatric trauma center, the condition of patients who suffer from trauma must meet specific anatomical and physiological criteria, which are regulated by the Regulation of the Minister of Health of 25 January 2016 on Trauma Centers for Children, with later changes. [8, 9, 21] At the same time, it should be emphasised that there is a study showing that the vast majority of Polish emergency departments do not meet the requirements imposed by state regulations. [22]

It is worth noticing that admission criteria for adults and children differ in several respects, with the paediatric criteria being more extensive. [9] Quite essential is also the addition of the third category to the paediatric patient criteria. It is suggested that the criteria for the adults in the Polish system might be too narrow, compared to CDC guidelines for field triage, for example, and so they may cause therefore quite substantial under-triage. [23] On the other hand, the data from our center may suggest that quite often patients qualified for admission to the PTCs don’t need prolonged transportation and care in such a highly specialised center.

The evaluation of the functionality, effectiveness, and eventually, the profitability of Polish centers is, at the moment, almost impossible to conduct. Obtaining the national information in the form of the screening of patient data from facilities with trauma centers based on the reported diagnoses seems to be the only possible way to get the necessary knowledge. This, however, leads to the incompatibility of pre-hospital and hospital data, since the patients are admitted to trauma centers because of criteria that determine their condition or circumstances at the site of the event, and not because of the final diagnosis. As an example, we can mention the Warsaw facility, from which data we have conducted detailed analysis, where according to the internal documentation system there were 32 patients admitted to the trauma center and according to data from the National Health Fund there were just 17. Due to this discrepancy, it should be noted that the summary presented above might be different from the data taken straight from the hospitals, but unfortunately, we were not able to directly obtain the data from the facilities. The lack of such a data collection system and the negative impact of such lack on patient care have also been highlighted in other research papers published to date. [5, 7]

The fact we found quite concerning is also the size of the zones, which are outside of the reachable distance from the Trauma Centers. It has been proven in the literature that the outcomes of treatment of traumatic patients are better inside 60 min driving time compared to outside of 60 min driving time from a PTC. [24] It does not mean that there are no emergency departments in the aforementioned areas, but it is hard to evaluate how well these hospitals are doing in assisting trauma patients and whether they would benefit from upgrading them to paediatric trauma centers.

Furthermore, if there was a pre-hospital death cases analysis system, we could assess whether, for example, a substantial number of patients had died due to or during prolonged transports caused by the excessive distance of the incident from the trauma centers. It has been suggested that trauma patients benefit greatly from being treated in PTC, rather than in smaller centers, but such analysis could give us a useful indication whether it would be better to transport the patients to the nearest emergency department, or to take the time for their transportation to the trauma center. [24, 25]

All things considered, we have concluded that there are no collective registers for paediatric trauma patients in Poland, that no one monitors the number of patients, their condition, and the results of short-term and long-term treatment. As a result, it is not possible to establish the exact trauma mortality among paediatric patients, the outcomes of their treatment, and the long-term morbidity of trauma patients.

Moving on to the data from the Warsaw PTC, we have noticed that one of the most common injuries in the paediatric population is head trauma, which is confirmed by the high percentage of neurosurgical interventions in these patients, as was found in both national data and our center data. What is more, based on the analysis of criteria admission, it seems that patients are often mistakenly classified to PTC, since only a minority of admitted patients fulfill the criteria. However, we have also observed that there was a higher incidence of surgical interventions in the non-criteria fulfilling group, and a substantial number of patients who did fulfill the anatomical criteria, without fulfilling physiological or additional ones. On the other hand, the patients who have met the criteria to be admitted to our trauma center had longer hospitalisation times and higher ICU admission rates [Table 2]. It can mean that, while the qualification criteria for the trauma center define a patient in a more serious condition or involved in a more serious accident, the majority of admitted patients could be treated in less specialised facilities, located closer to the site of the event. Another aspect worth considering is whether the additional criteria need some changes, as most of the children admitted under this category were patients requiring shorter and less intensive care than, for example, those from the car strike group.

As a result, it turns out that during the first year of the PTCs operation in Poland, a definite minority of patients were admitted due to a very poor general condition following the injury. This could either mean that there were patients meeting the admission criteria who were not accepted through the appropriate procedure, or perhaps the criteria for admission to a trauma center should be broaden, as suggested above. Finally, it could also mean that in 2019 there were few seriously injured paediatric patients. It would be extremely helpful if we could carry out such a detailed analysis for all 8 paediatric trauma centers currently operating in Poland.

## Conclusions

- From the available data, it can be inferred that the main cause of hospitalisation in paediatric trauma centers in 2019 in Poland was head injury and, for our center, the main reported incident was a fall from height.
- Our analysis has shown that the minority of patients hospitalised in the paediatric trauma centers met the formal criteria for their admittance.
- Although the number of surgical procedures performed in PTCs was higher in the group without fulfilled criteria, the ICU admission rate was higher, and the mean time of hospitalisation was longer in the group that fulfilled the criteria.
- Obtaining reliable data is made difficult or even impossible by the fact that there is no consistent database of paediatric trauma patients in Poland, as the National Health Fund collects data based on final diagnoses and not on preliminary criteria for admission.
- As a result, it is difficult to assess and evaluate the effectiveness and quality of the trauma care system for children in Poland, both in short-term and long-term outcomes, such as the morbidity of these patients over a period of several years, because no one is collecting data on the number of serious injuries in children, the condition of patients and their further treatment needs.
- So far, it is difficult to draw specific conclusions regarding the operation of PTCs in Poland, due to the small number of patients reported and the poor system of gathering complete clinical data. And that is why we believe that it would be profitable to create a better registration system which would allow conducting these assessments, as it could contribute to the continuous improvement of the paediatric trauma centers in Poland.
